# Novel composite drug delivery system as a novel radio sensitizer for the local treatment of cervical carcinoma

**DOI:** 10.1080/10717544.2017.1362676

**Published:** 2017-08-11

**Authors:** Shan Xu, Yu Ying Tang, Yan Xin Yu, Qin Yun, Jing Pin Yang, Heng Zhang, Qiuxia Peng, Xiaoyang Sun, Ling Lin Yang, ShaoZhi Fu, Jing Bo Wu

**Affiliations:** a Department of Oncology, The Affiliated Hospital of Southwest Medical University, Luzhou, Sichuan Province, China;; b Department of Oncology, MianYang Central Hospital, Mianyang, Sichuan Province, China;; c MianYang Central Hospital, Mianyang, Sichuan Province, China;; d Department of Oncology, The First People's Hospital of Guangyuan, Guangyuan, Sichuan Province, China

**Keywords:** Cervical cancer, cisplatin, paclitaxel, radiotherapy, thermosensitive hydrogel

## Abstract

In this study, we investigated *in vivo* radiosensitizing effects of a gel-based dual drug delivery system (DDS) (PECE/DDP + mPEG-PCL/PTX, or PDMP) in a cervical cancer model, and determined its possible mechanisms of action. A xenograft cervical cancer model was used to investigate the radio sensitization effect of PDMP. Mice underwent paclitaxel (PTX) + cisplatin (DDP), PECE, or PDMP treatment followed by single radiation doses ranging from 0 Gy to 20 Gy. Radio sensitization was analyzed by tumor regrowth delay (TGD). The sensitization enhancement ratio (SER) was calculated by the doses needed to yield TGD when using radiation treatment alone and when using radiation plus drug treatment. The impact of irradiation and drugs on TGD was determined, and an optimum radiation dose was chosen for further evaluation of radio sensitizing effects. The data showed that PDMP yielded the highest radio sensitization (SER was 1.3) and a radiation dose of 12 Gy was chosen for further investigation. PDMP + radiotherapy treatment was most effective in inhibiting tumor growth, prolonging survival time, decreasing expression of CD31, CD133, and aldehyde dehydrogenase 1 (ALDH1), inducing G2/M phase arrest, apoptosis, and expression of Ataxia telangiectasia mutated (ATM) and histone H2AX phosphorylation (γ-H2AX). Thus, our data indicated that PDMP is a promising anti-tumor and radio sensitization reagent for the treatment of cervical carcinoma.

## Introduction

In china, cases of cervical cancer show an increased prevalence in young patients (25–44 years of age), and both incidence and mortality rates are on the rise (Hong et al., 2013; Shuang et al., 2013). Because of its biological characteristics, cervical cancer cells undergo local invasion and local recurrence, therefore local treatment is general chosen as the primary treatment method (Waggoner, 2003). The majority of cervical cancer patients are treated with concurrent chemoradiation therapy, which improves both overall survival and disease-free survival (Denny, 2012). Cisplatin (DDP)-based chemotherapy is widely used and the combination of paclitaxel (PTX) and DDP is known to be effective in treatment of advanced and recurrent cervical cancer with response rates of 40–50% (Song et al., 2007; Mccormack et al., 2013; Mousavia et al., 2013). In addition, the use of PTX and DDP in combination with radiotherapy is a safe and effective treatment method for the treatment of locally advanced cervical cancer (Wang et al., 2015). Unfortunately, in tumor tissue, conventional chemotherapy-related drugs have failed to reach both the therapeutic dose and long retention period because of nonspecific interactions, which may cause adverse effects of the drug on the body, such as myelosuppression and gastrointestinal reactions (Rowinsky and Donehower, 1995; Cavaletti et al., 2011). In over 30% patients treatment had to be terminated early because of adverse effects of the drugs used (Hashemi et al., 2013). Therefore, a novel drug delivery system that results in a higher concentration of drugs in the tumor, an increased retention time, and reduced side effects is clearly warranted.

Due to their high local drug concentration and low levels of systemic toxicity, injectable *in situ*-forming hydrogels, such as poly(ethylene glycol)-poly(ɛ-caprolactone)-poly(ethylene glycol) (PEG-PCL-PEG, PECE) have been extensively studied as novel drug delivery systems (DDSs) in recent years (Fang et al., 2009; Na et al., 2012; Lin et al., 2014; Wu et al., 2014). Degradable polymers are of interest as DDSs because of their ability to increase water solubility and anti-tumor effects of hydrophobic chemotherapeutic agents, such as monomethyl poly(ethylene glycol)-poly(ε-caprolactone) (mPEG-PCL) (Gong et al., 2012; Wu et al., 2014). In our previous study, we created a novel *in situ* gel-forming hydrogel composite, PDMP, which is composed of mPEG-PCL/PTX micelles and a DDP-loaded PECE hydrogel (Wu et al., 2014). Prior to administration, the PDMP hydrogel composite is present as an aqueous solution, however once injected *in vivo*, the composite rapidly forms a gel. The PDMP hydrogel has shown to have great potential for the treatment of lung cancer. In this study, we used a Hela xenograft model to verify the anti-tumor effects of PDMP by evaluating *in vivo* tumor growth inhibition. In addition, we studied the radio sensitization effect of PDMP *in vivo* by using different radiation doses and explored the mechanism of radio sensitivity of PDMP.

## Materials and methods

### Murine tumor models and treatment

The preparation of PDMP hydrogel composites and methods for cell culture are as previously described (Wu et al., 2014). Specific details regarding these methods are described in the supplementary materials. A schematic diagram of the PDMP hydrogel complex is presented in Supplementary Figure S1.

Hela cells (1 × 10^6^/mL) were implanted in the right thigh of female BALB/c nude mice. The tumor volume was calculated according to the following formula: tumor size = (length) × (width)^2^/2. Two weeks after tumor implantation the tumor reached a volume of 150–200 mm^3^ at initiation of treatment.

To determine whether PDMP could enhance the radiation effect, 150 tumor-bearing mice were randomly assigned to one of the following five groups (*n* = 30 mice per group): (1) Control; (2) Blank thermosensitive hydrogel (PECE); (3) PTX (5 mg/kg) and DDP (2 mg/kg) intra-tumor injection (PTX + DDP(IT); (4) PTX (5 mg/kg) and DDP (2 mg/kg) intraperitoneal injection (PTX + DDP(IP); (5) PDMP hydrogel composite. The doses for PTX (5 mg/kg) and DDP (2 mg/kg) were chosen according to previous studies, which proved to have a beneficial effect in a mouse model of cervical cancer (Pisano et al., 2003; Wu et al., 2014). Next, each of the afore-mentioned groups were divided into foue subgroups: plus 0 Gy, 5 Gy, plus 10 Gy, plus 15 Gy, and plus 20 Gy (*n* = 6 mice per group). Mice were irradiated in a leg restraint box, which means that only their right tumor-bearing thigh received a local dose of radiation (Figure S2). During this phase, drugs were administrated via intraperitoneal injection 15–30 min before irradiation, whereas intratumoral injections of drugs were given within 15 min before irradiation (Yapp et al., 1998). In addition, the volume of PDMP was based on the body weight of mice and was between 100 and 200 µL. After irradiation, tumors were measured every two days. Tumor growth curves were constructed according to the Gompertz model using the following equation: *y* =* V*_0_*exp(k*(1 − exp(−a*X))), where *V*_0_ is the original tumor volume (150–200 mm^3^); *k* and *a*, coefficient; *X*, irradiation dose, and *y* tumor growth delay (TGD) time (Zhang et al., 2011). TGD was defined as the difference between the number of days required for tumor growth to reach four times its original volume (T4V0). Tumor growth was analyzed by the general linear model and optimal dose of radiation was used for further evaluation. More specially, 60 tumor-bearing mice were randomly assigned to one of the following five groups (*n* = 12): 1, RT; 2, PECE + RT; 3, PTX + DDP(IT)+RT; 4, PTX + DDP(IP)+RT; 5, PDMP + RT. The tumor was measured every two days and at 100 days after treatment. Three mice per group were sacrificed after two days of treatment and tumor tissue was harvested for γ-H2AX evaluation by immunohistochemistry and Ataxia telangiectasia mutated (ATM) analysis by Western blot. In addition, three mice per group were sacrificed after 10 days of treatment, and tumor tissue was harvested for flow cytometry and immunohistochemical analysis. The remaining mice were used for observation of tumor growth and survival time. All animal care and experimental procedures were conducted according to Institutional Animal Care and Use guidelines.

### Toxicity assessment

Possible side effects were indicated by observation of body weight, appetite, diarrhea, life span, and behavior. After mice were sacrificed, liver, lungs, kidney, spleen, and heart were harvested for hematoxylin and eosin (H&E) staining. H&E-stained slides were observed by two pathologists in a blinded manner.

### Immunohistochemistry

Tumors were fixed in 10% neutral-buffered formalin solution, embedded in paraffin, and 4 μm thick sections were cut for immunohistochemical analysis. Sections were stained with antibodies directed against γ-H2AX, CD31, and CD133, and were performed according to the manufacturer’s instructions (Bioworld Technology, Nanjing, China). Images were taken using an optical microscope (Olympus, Tokyo, Japan). In each tumor section, the number of γ-H2AX and CD133 positive cells was calculated in five randomly selected areas (at 400 × magnification) as the number of positive cells/total cells. Microvessel density (MVD) of tumor tissues was calculated as the mean value of CD31-positive microvessels in five randomly selected areas (at 400 × magnification).

### Western blot analysis

Tumor samples were homogenized, and centrifuged at high speed for 10 min at 4 °C. The supernatant was collected and used for bicinchoninic acid (BCA) protein determination (BCA Protein Assay Reagent; Thermo Fisher Scientific, Rockford, IL, USA) and Western blot analysis. A total of 40 μg of protein was separated on a 4–20% Tris-glycine gel and transferred to 0.45 μm nitrocellulose membranes. Membranes were blocked with 5% nonfat milk in Tris-buffered saline solution. After blocking, membranes were incubated overnight at 4 °C with the primary antibody (anti-ATM monoclonal antibody MAT3–4G10/8, Sigma). Next, membranes were washed three times for 10 min with Tris-buffered saline solution and incubated with a peroxidase-conjugated secondary antibody for 1 h under shaking at room temperature. After incubation, membranes were washed three times with Tris-buffered saline solution for 10 min and proteins were visualized using chemiluminescence. Glyceraldehyde-3-phosphate dehydrogenase (GAPDH) served as a control. Signals were quantified using ImageQuant 5.0 software (Molecular Dynamics, Sunnyvale, CA, USA).

### Flow cytometry

A single-cell suspension of 1 × 10^6^ cells/mL was prepared from isolated tumor tissue, and 10 mL of this suspension was aliquoted into individual test tubes. A total of 5 mL of Annexin Vefluorescein isothiocyanate (FITC) and 5 mL of propidium iodide was added to each test tube according to the desired analysis. Cells were incubated in an ice bath for 15 min in the dark, followed by addition of 400 mL ice-cold 1 × binding buffer and gentle mixing. Next, cells were analyzed using an Epics XL flow cytometer (Beckman Coulter, Miami, FL, USA).

### Statistical analysis

Statistical analysis was performed using SPSS 17.0 software. Comparison of the mean values was performed by Student’s *t*-test and one-way analysis of variance (ANOVA). Comparison of tumor growth was performed using repeated-measures ANOVA. The impact of irradiation and drugs on tumor growth was analyzed by the general linear model, and differences among groups were compared by the Student–Newman–Keuls test. Data were considered significantly different when *p* < .05. The sensitization enhancement ratio (SER) was used to evaluate the radiation effect of PDMP. The biological endpoint was defined as a TGD of 20 days for *in vivo* experiments, and SER was radiation dose required to reach the endpoint in the irradiation alone versus irradiation dose plus combination groups. Moreover, we ruled out the possibility that the effects of the drugs were additive to those of radiation treatment alone. The actual radio sensitization was calculated using the following equation: TGDrad = (TGD per treatment group plus irradiation) – (TGD per treatment group without irradiation).

## Results

### *In vivo* tumor radio sensitization testing

PDMP and PECE hydrogels were successfully synthesized according to methods previously reported and are further described in the supplementary materials (Wu et al., 2014).

In this study, a xenograft cervical cancer model was used to evaluate the radio sensitization of PDMP hydrogels. A single dose of 0, 5, 10, 15, or 20 Gy was given to the control, PTX + DDP (IP), PTX + DDP (IT), PECE, and PDMP groups. The TGD in cervical cancer after different treatments is shown in Table S1 and Figure S5. Treatment of PDMP alone yielded a TGD of eight days, which is significantly different from the PTX + DDP(IT) group (4.7 days, *p* < .05), PTX + DDP(IP) group (1.3 days, *p* < .05), PECE group (0 days, *p* < .05), and control group (0 days, *p* < .05).

The radio sensitization data of each group is shown in Table 1. Our data showed that an increase in irradiation dose gradually reduced tumor growth and extended TGD in all treatment groups. PDMP + RT groups, when using 5, 10, 15 and 20 Gy, produced significantly longer TGD compared to other irradiation groups (in all cases *p* < .05).

**Table 1.** TGDrad in cervical cancer after diverse treatment groups plus different irradiation dose.

Groups	0 Gy	5 Gy	10 Gy	15 Gy	20 Gy
RT	0	6.7	12.7	18.7	24.7
PECE + RT	0	6.7	12	17.3	22.7
PTX + DDP(IT) + RT	4.7	6	12	19.9	26
PTX + DDP(IP) + RT	1.3	7.4	12	19.4	24.7
PDMP + RT	8	6.7	18	24	36.7

TGDrad: TGDrad = (TGD in each treatment group plus irradiation) – (TGD in each treatment groups without irradiation); PTX: paclitaxel; DDP: cisplatin; PDMP: mixing MPEG-PCL/PTX micelles with DDP-loaded PECE hydrogel; IP: intraperitoneal injection; IT: intra-tumor injection.

**Figure 1.**
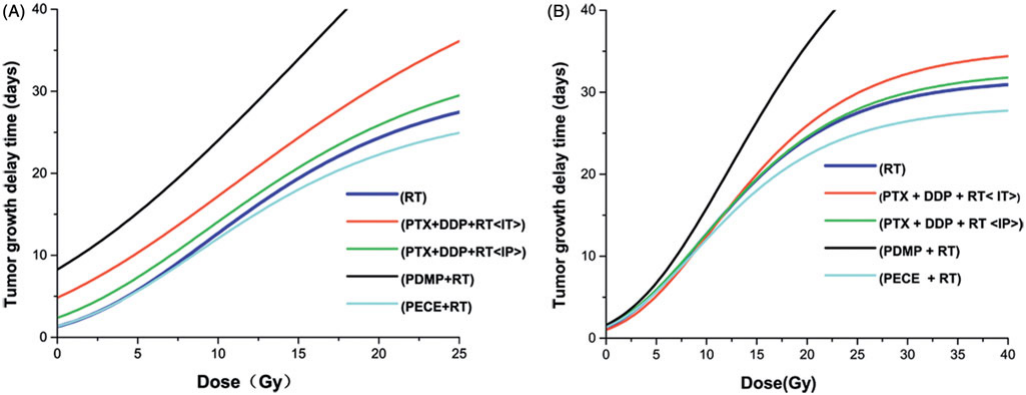
Dose–response curves based on TGD and TGDrad. (A) Dose–response curves based on TGD; (B) Dose–response curves based on TGDrad; TGDrad, TGDrad = (TGD per treatment group plus irradiation) – (TGD per treatment group without irradiation); PTX: paclitaxel; DDP: cisplatin; PDMP: mixing mPEG-PCL/PTX micelles with DDP-loaded PECE hydrogels; IP: intraperitoneal injection; IT: intratumoral injection.

Dose–effect curves were generated for RT, PECE + RT, PTX + DDP (IP)+RT, PTX + DDP(IT)+RT, and PDMP + RT groups (Figure 1). Curves were nearly parallel when TGD was between 10 and 20 days (Figure 1(A)). Therefore, a TGD of 20 days was chosen as a biological endpoint. To achieve a TGD of 20 days, irradiation doses of 15.5, 11.9, 14.4, 17.0 and 7.8 Gy were required for RT, PTX + DDP (IT)+RT, PTX + DDP (IP)+RT, PECE + RT and PDMP + RT groups, respectively. The SER was 1.98 (15.5/7.8) for the PDMP + RT treatment group, 1.3 (15.5/11.9) for the PTX + DDP (IT)+RT group, 1.07 (15.5/14.4) for the PTX + DDP (IP)+RT group, and 0.9 (15.5/17) for the PECE + RT group.

In addition, we ruled out the possibility that the effects of the drugs were largely additive to those of radiation. The actual radio sensitization was calculated by TGDrad (Figure 1(B)). To achieve a TGDrad of 20 days, 15.5, 14.9, 15.4, 17.1 and 11.9 Gy were required for RT, PTX + DDP (IT)+RT, PTX + DDP (IP)+RT, PECE + RT, and PDMP + RT groups, respectively. The SER was 1.3 (15.5/11.9) for the PDMP + RT treatment group, 1.04 (15.5/14.9) for the PTX + DDP (IT)+RT group, 1.0 (15.5/15.4) for the PTX + DDP (IP)+RT group, and 0.9 (15.5/17.1) for the PECE + RT group.

### *In vivo* tumor inhibition and radio sensitization test when combined with 12 Gy

**Figure 2.**
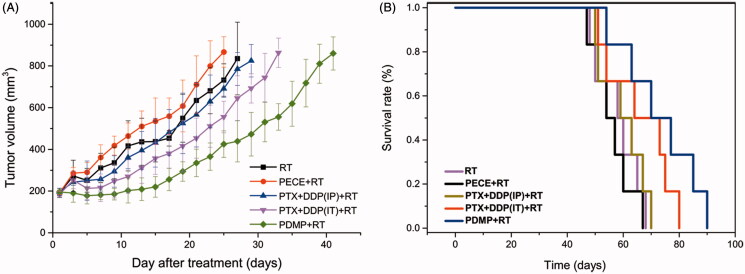
Treatment with PDMP + RT inhibited tumor growth in a subcutaneous HeLa model. (A) Suppression of tumor growth after PDMP + RT treatment in mice; (B) mouse survival curves per group; PTX: paclitaxel; DDP: cisplatin; PDMP: mixing mPEG-PCL/PTX micelles with DDP-loaded PECE hydrogels; IP: intraperitoneal injection; IT: intratumoral injection.

As shown in Figure 1, an increase in irradiation dose gradually slows down tumor growth and extended TGDrads in all treatment groups. Treatment with PDMP showed significant radio sensitivity compared to other treatment groups when a TGDrad of 20 days was chosen. To confirm the radio sensitization of PDMP, 12 Gy was chosen for further investigation. Another reason for choosing a dose of 12 Gy was that the incidence of severe side effects significantly increased in all treatment groups when the doses of irradiation were too high (15 Gy or 20 Gy), such as radiation edema, radiodermatitis, radioactive rectitis, emaciation. Moreover, several mice did not survive (Figure S6). Thus, a single dose of 12 Gy was given to mice in control, PTX + DDP (IP), PTX + DDP (IT), PECE and PDMP treatment groups. Tumor growth curves are shown in Figure 2(A), whereas the calculated TGD is shown in Table 2. Data indicated that treatment with PDMP + RT had the greatest efficacy in tumor growth inhibition compared with other groups (*p* < .05 in all cases). In addition, the median survival time of mice shows similar results (Figure 2(B) and Table S2). The PDMP + RT treatment group showed a survival of 73 days, which was significantly longer compared to that of mice treated with PTX + DDP (IT)+RT (66 days, *p* < .01), RT (58 days, *p* < .01), and other groups (*p* < .01 in all cases). No significant differences were observed in tumor growth and survival time between treatment with RT or PECE + RT (*p* > .05 in all cases). These results suggested that PDMP + RT was effective in inhibiting tumor growth as well as in prolonging survival time of tumor-bearing mice.

**Table 2.** Tumor growth delay (in days) in cervical cancer after diverse treatment groups plus 12 Gy irradiation.

Groups	T4v0 (days)	TGD (days)
RT	26.2	14
PECE + RT	24	13.8
PTX + DDP(IP)+RT	26.6	14.4
PTX + DDP(IT)+RT	31.4	19.2
PDMP + RT	39.8	27.6

T4v0: number of days needed for tumor growth to reach four times the original volume; TGD: tumor growth delay, which in days was defined as the difference between T4V0 of treated tumors compared with untreated tumors; PTX: paclitaxel; DDP: cisplatin; PDMP: mixing MPEG-PCL/PTX micelles with DDP-loaded PECE hydrogel; IP: intraperitoneal injection; IT: intra-tumor injection.

### Immunohistochemistry

The effect of deoxyribonucleic acid (DNA) damage in experimental groups can be determined by assessing the expression of tumor-associated γ-H2AX. When compared with other groups, an increased number of γ-H2AX positive cells was observed in tumor tissue derived from mice treated with PDMP + RT (Figure 4). Quantitative analysis of γ-H2AX positive cells in the different treatment groups indicated the following order:

PDMP + RT > PTX + DDP(IT)+RT > PTX + DDP(IP)+RT > PECE + RT > RT (Figure 5(A) and Table S3). The number of γ-H2AX positive cells in tumor tissue derived from PDMP + RT-treated mice (96.25 ± 4.78%) was significantly higher compared to that of PTX + DDP(IT)+RT-treated mice (76.14 ± 5.76%, *p* < .05), RT-treated mice (70.88 ± 4.08%, *p* < .05) and mice in other treatment groups (*p* < .05 in all cases). No significant differences were observed between mice in RT and PECE + RT groups (*p* > .05 in all cases). These results indicated that combination treatment of PDMP and RT significantly increased the number of DNA double-strand breakages.

**Figure 3.**
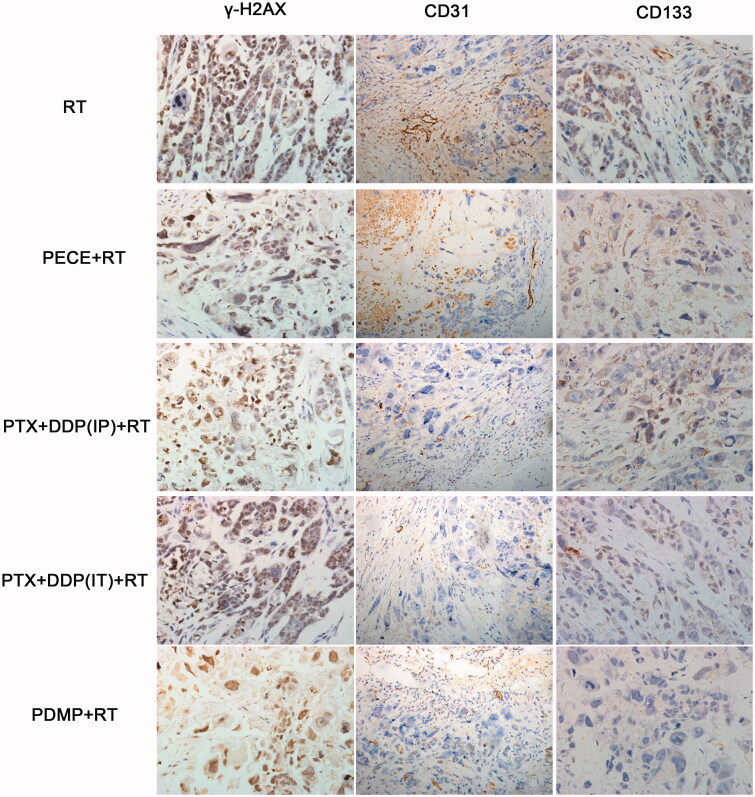
Immunohistochemical analysis of γ-H2AX, CD133, and CD31 in a mouse xenograft model. PTX: paclitaxel; DDP: cisplatin; PDMP: mixing mPEG-PCL/PTX micelles with DDP-loaded PECE hydrogels; IP: intraperitoneal injection; IT: intratumoral injection.

**Figure 4.**
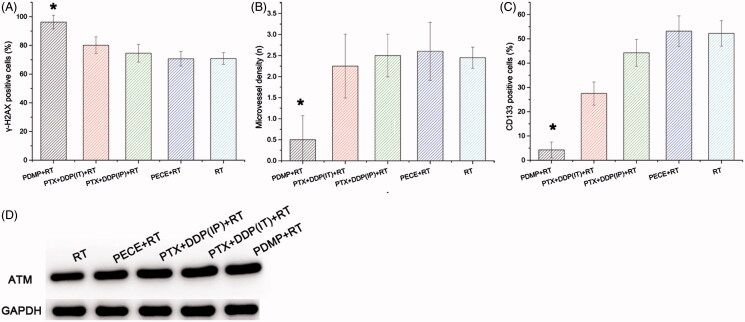
Quantitative analysis of γ-H2AX, CD133, and CD31 and the expression of ATM in xenografts from mice in various treatment groups. (A) quantitative analysis of γ-H2AX in mouse xenografts in various treatment groups; (B) quantitative analysis of CD133 in mouse xenografts in various treatment groups; (C) quantitative analysis of CD31 in mouse xenografts in various treatment groups; (D) expression levels of ATM in mouse xenografts in treatment various groups; PTX: paclitaxel; DDP: cisplatin; PDMP: mixing mPEG-PCL/PTX micelles with DDP-loaded PECE hydrogels; IP: intraperitoneal injection; IT: intratumoral injection.

The expression of CD31, an endothelial cell surface molecule that can be used to investigate MVD, was determined by immunohistochemistry (Figure 3). The MVD in the different treatment groups was determined as follows: PDMP + RT < PTX + DDP(IT)+RT < RT < PTX + DDP(IP)+RT < PECE + RT (Figure 4(B) and Table S4). The MVD of tumor tissue derived from mice in the PDMP + RT group (0.5 ± 0.57) was significantly lower compared to those of mice in the PTX + DDP(IT)+RT group (2.25 ± 0.76, *p* < .05), the RT group (2.45 ± 0.25, *p* < .05), and other groups (*p* < .05 in all cases). Thus, these data showed that in the PDMP + RT group, there is a significant decrease in MVD.

The effect of tumor proliferation was assessed by immunohistochemical detection for CD133 (Figure 3), which has been reported as a molecular marker of cancer stem cells (CSC) (Zhou et al., 2010). Regarding the percentage of CD133 positive cells in the different treatment groups, the following order was identified: PDMP + RT < PTX + DDP(IT)+RT < PTX + DDP(IP)+RT < RT < PECE + RT (Figure 4(C) and Table S5). A reduction in CD133 positive cells was observed in tumor tissues derived from mice in the PDMP + RT group (4.25 ± 3.27) when compared with mice in the PTX + DDP(IT)+RT group (27.5 ± 4.76%, *p* < .05), RT group (52.25 ± 5.25%, *p* < .05), and other groups (*p* < .05 in all cases). Together, these data showed that combination treatment of PDMP and RT inhibited CSC, which may contribute to the inhibition of tumor growth.

### Western blot analysis

In irradiated cells, ATM is one of the earliest and most sensitive responses to DNA damage (Matsuoka et al., 2007). To further investigate the levels of DNA damage in our study, the expression of ATM protein was evaluated by Western blot analysis (Figure 4(D)). Levels of ATM in the different treatment groups were identified as follows: PDMP + RT > PTX + DDP(IT)+RT > PTX + DDP(IP)+RT > PECE + RT > RT. These results indicated that combination treatment of PDMP and RT increased the frequency of DNA double-strand breakages.

### Flow cytometry

To identify the mechanism of action that is responsible for the differences observed in tumor growth, we analyzed cell cycle redistribution, apoptosis, and CSC by flow cytometry (Table S6).

**Figure 5.**
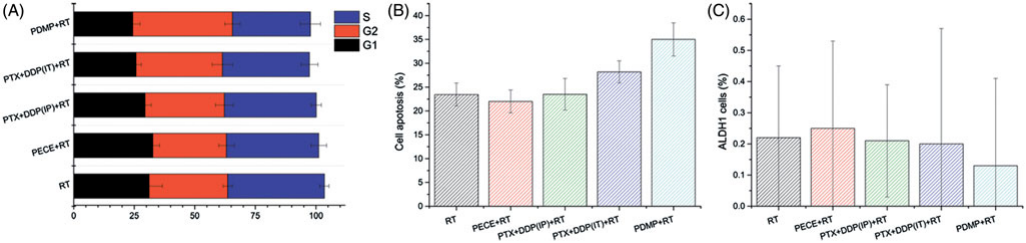
Flow cytometry analysis of tumor tissue from mice that received different treatments. (A) quantitative analysis of the percentage of cells in G1, S, G2/M phase in mouse xenografts in various treatment groups; (B) quantitative analysis of the percentage of apoptosis in mouse xenografts in various treatment groups; (C) quantitative analysis of the percentage of ALPH1 in xenografts from mice in various groups; PTX: paclitaxel; DDP: cisplatin; PDMP: mixing mPEG-PCL/PTX micelles with DDP-loaded PECE hydrogels; IP: intraperitoneal injection; IT: intratumoral injection.

Figure 5(A) showed that all treatment groups lead to high proportion of cells in phase G2. However, no significant differences were found in the percentage of cells in the S phase (*p* > .05 in all cases). PDMP + RT treatment resulted in an increased number of cells in the G2 phase (41.12 ± 3.19%) compared to the PTX + DDP(IT)+RT treatment group (35.9 ± 4.27%, *p* < .05), RT group (32.66 ± 3.69%, *p* < .05), and other treatment groups (*p* < .05 in all cases, Figure S8).

The induction of apoptosis was also assessed by using flow cytometric analysis. As shown in Figure 5(B), the PDMP + RT treatment group showed a significant increase in the percentage of cell death (36.25 ± 3.5%) when compared to the PTX + DDP(IT)+RT treatment group (28.25 ± 2.36%, *p* < .05), RT group (24.75 ± 3.65%, *p* < .05), and other groups (*p* < .05 in all cases, Figure S9).

To further determine whether PDMP treatment inhibited cell proliferation, expression of ALPH1, a Cancer stem cells marker in HeLa cells, was evaluated by flow cytometry. Figure 5(C) shows that PDMP + RT treatment reduced the percentage of ALPH1 (0.13 ± 0.28%) when compared to PTX + DDP(IT)+RT treatment (0.2 ± 0.37%), RT treatment (0.22 ± 0.23%%) and other treatments. However, no significant differences were observed among these groups (*p* > .05 in all cases, Figure S10).

### *In vivo* toxicity in PDMP-treated mice

Since previous studies have confirmed severe irreversible side effects of PTX and DDP treatment, we investigated changes in body weight and other side effects in mice treated with PTX + DDP or PDMP. No differences in body weight were observed in PDMP-treated mice compared with mice in other groups without irradiation. However, only a slight decrease in body weight was observed after irradiation treatment, however after a short period of time (5 or 6 days) weight returned to normal. After mice were sacrificed, liver, lungs, kidney, spleen, and heart were harvested and H&E staining was performed. Slides were evaluated in a blinded manner by two pathologists, and it was found that mice treated with PECE and PDMP demonstrated mild liver toxicity (ballooning degeneration). We speculated that this may be related to degradation of the polymer. In addition, mice that underwent irradiation demonstrated similar liver toxicity (Figure S7). However, in other organs no signs of toxicity were observed. Moreover, no adverse effects were observed among groups, including changes in appetite, feeding, or behavioral change.

## Discussion

Cervical cancer is the second most common type of cancer in females worldwide, and is associated with local invasion and recurrent tumors (Yu et al., 2017). Several studies have demonstrated that local treatment of cervical cancer may reduce parametrial infiltration and destroy micrometastases, thereby decreasing relapse and metastasis (Thomas, 1999, 2000). It is well known that in cervical cancer a tight linkage exists between locoregional recurrence and distant metastasis. Concurrent cisplatin-based chemotherapy plus radiotherapy is the most frequently chosen treatment regimen for cervical cancers. Although significant progress has been made in treating cervical cancer, chemotherapy can be associated with significant signs of nausea, hematologic toxicity, and myelosuppression that may offset the therapeutic benefits of chemotherapy. Therefore, novel formulations of chemotherapy drugs have become a hotspot in the treatment of cervical cancer. Injectable *in situ*-forming hydrogels have been extensively studied in novel DDSs, and have shown advantages in improving the concentration of drugs in the tumor to achieve longer retention and reduced side effects (Wu et al., 2014). In addition, the physical position and anatomical structure of the cervix is extremely suitable for the use of situ-forming hydrogel. Thus, PDMP hydrogels may have great potential for in situ treatment of cervical cancer.

In our previous study (Wu et al., 2014), we investigated PDMP hydrogels in *in vitro* drug release studies by dialysis, *in vitro* cytotoxicity assays, and *in vivo* anti-tumor efficacy in a xenograft lung cancer model. Our results demonstrated that PDMP had a slower drug release rate (more than 14 days), minor toxicity, and was effective in inhibiting tumor growth and prolonged survival in a xenograft lung cancer model.

The central aim of this study was to further confirm the anti-tumor effect of PDMP hydrogels, and to study its effects in combination with radiation as a radio sensitizer. We established a xenograft human cervical model in nude mice in which we investigated the radio sensitization properties of PDMP. A general linear model was used to analyze the impact of drugs and irradiation by using a range of radiotherapy doses. The data indicated that PDMP hydrogels have great tumor inhibiting properties, and PTX + DDP (IT) and PDMP enhanced radio sensitization compared with irradiation alone. Moreover, PDMP has a stronger radio sensitization effect compared to PTX + DDP (IT) (SER of 1.30 vs 1.04). This may be due to the fact that PDMP caused sustained slow release of PTX and DDP when combined with radiation. First, DDP and PTX are radiation sensitizers and have been widely used in combination with radiation therapy in cancer treatment. In addition, our previous study (Wu et al., 2014) demonstrated that *in vitro* mPEG-PCL/PTX slows down the release of PTX compared to free PTX (14 days vs. 4 days). Lei et al. (2012) reported that the release rate of PTX from PTX-PECE *in vitro* remained steady for up to 20 days and that *in vivo* over a period of 10 days, the local drug concentration in mice that received PTX-PECE treatment was higher compared to that of free PTX. Therefore, we mixed mPEG-PCL/PTX micelles with DDP-loaded PECE hydrogels, named PDMP. Due to its release function, PDMP increased PTX and DDP exposure time in tumors, similar as when using a drug delivery pump. After irradiation of tumors, the PTX and DDP deposited in tumors served as radio sensitizers. Free PTX and DDP, however, were quickly excreted from the body, therefore maintaining a low concentration in the tumor for a relatively short period of time.

This *in vivo* study indicated that treatment with PDMP + RT showed significant radio sensitivity compared to other treatment groups when a TGDrad of 20 days was chosen and the ideal dose of radiotherapy was 12 Gy. Thus, 12 Gy was chosen for further investigation. In our study, several observations were made to evaluate the therapeutic activities of RT treatment combined with PDMP hydrogel composites. Mice in the PDMP + RT group showed the greatest efficacy in TGD, which resulted in a higher survival rate compared to other groups. To investigate the mechanism of enhanced anti-tumor effect of PDMP treatment in combination with RT, animals were sacrificed after 2 and 10 days of treatment (Gong et al., 2009), and tumor tissue was harvested for immunohistochemical analysis, Western blot analysis and flow cytometry for markers, including γ-H2AX, CD31, CD133 and ATM.

Given that PTX and DDP could induce tumor cell apoptosis and affect cell cycle distribution, we investigated the radio sensitivity and anti-tumor effect of PDMP, cell cycle, and apoptosis by flow cytometry. Our results indicate an increasing number of cells in the G2 phase in the PDMP + RT treatment group. This result may imply that the synergistic effect of PDMP combined with RT may increase the cell ratio of the G2/M phase, resulting in a higher radio sensitivity of tumor cells to radiation. Apoptosis is a type of programed cell death, involving numerous pathological and physiological processes, and plays a vital role in the enhancement of drug activity and radiation treatment. A significant increase in the induction of apoptosis was observed in the PDMP + RT treatment group as compared to other treatment groups, indicating that PDMP + RT treatment contributes to the inhibition of tumor growth.

DNA is the major target of ionizing radiation and includes base damage, sugar damage, single-strand breaks (SSBs) and double-strand breaks (DSBs), of which DSBs induce histone H2AX phosphorylation, leading to cell death. In our study, the expression of γ-H2AX was investigated as a biomarker for DSBs. The PDMP + RT treatment group exhibited significantly higher expression of γ-H2AX compared to other groups. ATM also plays a key role in detecting DNA DSBs and in coordinating DNA repair, cell cycle arrest, and induction of apoptosis (Burma et al., 2001). Therefore, we monitored the expression of ATM to further confirm DNA DSBs by Western blot analysis. In our study, the highest levels of ATM were found in the PDMP + RT treatment group. Therefore, the radio sensitivity of PDMP may be due to enhanced X-ray-induced DSBs.

Angiogenesis is a key feature of tumor growth and progression, therefore, tumor vascular-targeting therapy has become a hotspot in the treatment of various tumors (Roudsari & West, 2016). In this study, we assessed the anti-angiogenesis activity of PDMP treatment by immunohistochemical analysis of CD31, an endothelial cell surface molecule that can be used to determine MVD. Our data showed that treatment with PDMP + RT resulted in the lowest MVD compared to other groups. It is well-known that treatment with PTX and DDP inhibited tumor angiogenesis and that PDMP treatment improved anti-angiogenic activity by cumulating PTX and DDP release, especially when used in combination with radiotherapy.

Cancer stem cells are essential for tumor growth and metastatic spread, and represent an important prognostic indicator. In our study, stem cell-related marker CD133 was evaluated by immunohistochemistry. When compared to other treatment groups, treatment with PDMP + RT resulted in the lowest number of CD133 positive cells. Previous studies have shown that ALDH1 has been implicated in cancer pathogenesis, and was used as a CSC marker (Yao et al., 2015). Our data showed that the PDMP + RT treatment group showed the lowest ALDH1 expression. These results were consistent with CD133 expression, suggesting that the anti-tumor and radio sensitization effects of PDMP may be due to the inhibition of cancer stem cells, which contribute to the inhibition of tumor growth.

To investigate the *in vivo* cytotoxicity of PDMP, H&E staining was performed to determine the effect on liver, lung, kidney, spleen, and heart. As also indicated in our previous study, PDMP was well-tolerated and did not show any toxic effects.

## Conclusions

In summary, the data presented in our study are consistent with previous reports, which showed that PDMP has excellent anti-tumor effects, and are well tolerated by the mice in the model used. In addition, we demonstrated that PDMP has a radio sensitizing effect in a Hela xenograft model. More importantly, we have evaluated the mechanism of action underlying the radio sensitizing effect and showed that PDMP increased the cell ratio of the G2/M phase, thereby enhancing X-ray-induced DSBs, inhibiting tumor angiogenesis, inhibiting cancer stem cells, and significantly increasing the induction of apoptosis. In conclusion, PDMP is a promising anti-tumor and radio sensitization reagent for local treatment of cervical carcinoma.
